# 1-(3-Mesityl-3-methyl­cyclo­butyl)-2-phenoxy­ethanone

**DOI:** 10.1107/S1600536810003910

**Published:** 2010-02-06

**Authors:** Murat Koca, Cumhur Kirilmiş, Cengiz Arici

**Affiliations:** a Department of Chemistry, Faculty of Arts and Science, Adiyaman University, 02040 Adıyaman, Turkey; b Department of Engineering Physics, Hacettepe University, Beytepe 06800, Ankara, Turkey

## Abstract

In the title compound, C_22_H_26_O_2_, the cyclo­butane ring is puckered, with a dihedral angle of 24.97 (9)° between the two C_3_ planes. In the crystal, inter­molecular non-classical C—H⋯O inter­actions between the methyl­cyclo­butyl CH group and the O atom of the phen­oxy group are found.

## Related literature

For related cyclo­butanes, see: Çukurovali *et al.* (2005 ▶); Dinçer *et al.* (2004 ▶); Kirilmiş *et al.* (2005*a* ▶,*b* ▶); Sari *et al.* (2002 ▶, 2004 ▶). For the anti-inflammatory and anti-depressant activity of three-substituted cyclo­butane acid derivatives, see: Roger *et al.* (1977 ▶); Gerard (1979 ▶); Sawhney *et al.* (1978 ▶); Brown *et al.* (1974 ▶); for anti-microbial activity, see: Suziki *et al.* (1979 ▶); for anti-parasitic activity, see: Slip *et al.* (1974 ▶), for herbicidal activity, see: Foerster *et al.* (1979 ▶) and for their liquid-crystal properties, see: Dehmlow & Schmidt (1990 ▶).
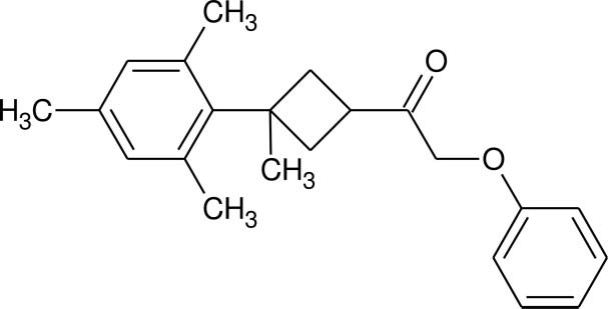


## Experimental

### 

#### Crystal data

C_22_H_26_O_2_
*M* *_r_* = 322.43
Triclinic, 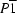

*a* = 8.5884 (12) Å
*b* = 10.1725 (11) Å
*c* = 11.1018 (12) Å
α = 82.364 (4)°
β = 68.170 (3)°
γ = 86.235 (2)°
*V* = 892.24 (19) Å^3^
*Z* = 2
Mo *K*α radiation
μ = 0.08 mm^−1^
*T* = 100 K
0.42 × 0.33 × 0.24 mm

#### Data collection

Nonius KappaCCD diffractometer
Absorption correction: multi-scan (*SADABS*; Bruker–Nonius, 2002 ▶) *T*_min_ = 0.969, *T*_max_ = 0.982
23641 measured reflections
3921 independent reflections
2968 reflections with *I* > 2σ(*I*)
*R*_int_ = 0.054

#### Refinement

*R*[*F* ^2^ > 2σ(*F* ^2^)] = 0.047
*wR*(*F* ^2^) = 0.129
*S* = 1.07
3921 reflections
217 parameters
H-atom parameters constrained
Δρ_max_ = 0.32 e Å^−3^
Δρ_min_ = −0.25 e Å^−3^

### 

Data collection: *COLLECT* (Bruker–Nonius, 2002 ▶); cell refinement: *EVALCCD* (Bruker–Nonius, 2002 ▶); data reduction: *EVALCCD* program(s) used to solve structure: *SHELXS97* (Sheldrick, 2008 ▶); program(s) used to refine structure: *SHELXL97* (Sheldrick, 2008 ▶); molecular graphics: *PLATON* (Spek, 2009 ▶); software used to prepare material for publication: *SHELXL97*.

## Supplementary Material

Crystal structure: contains datablocks global, I. DOI: 10.1107/S1600536810003910/rk2189sup1.cif

Structure factors: contains datablocks I. DOI: 10.1107/S1600536810003910/rk2189Isup2.hkl

Additional supplementary materials:  crystallographic information; 3D view; checkCIF report

## Figures and Tables

**Table 1:** Hydrogen-bond geometry (Å, °)

*D*—H⋯*A*	*D*—H	H⋯*A*	*D*⋯*A*	*D*—H⋯*A*
C10—H10*A*⋯O1^i^	0.99	2.44	3.419 (3)	172 (2)

Symmetry code: (i) 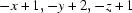.

**Fig. 1.**
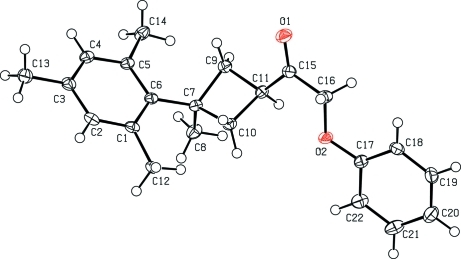
View of the title molecule with the atom numbering scheme. The displacement ellipsoids are drawn at the 50% probability level. H atoms are shown as a small circles of arbitrary radii.

**Fig. 2.**
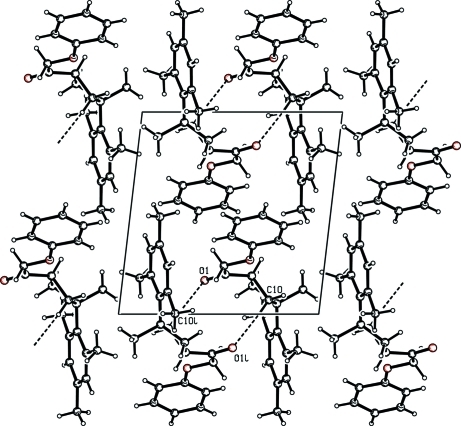
A packing diagram of title compound with the intermolecular H bonds (dashed lines). Symmetry code: (i) -x+1, -y+2, -z+1.

## supplementary crystallographic information

### Comment

It is well known that three-substituted cyclobutane acid derivatives exhibit anti-inflammatory and anti-depressant activities (Roger *et al.*, 1977; Gerard, 1979) and liquid-crystal properties (Dehmlow & Schmidt, 1990), moreover their of various thiazoles derivatives showed herbicidal (Foerster *et al.*,  1979), anti-inflammatory (Sawhney *et al.*, 1978; Brown *et al.*,  1974), anti-microbial (Suziki *et al.*,  1979) or anti-parasitic activity (Slip *et al.*,  1974). Substituted cyclobutane contained similar structures, have been reported in the recent years (Kirilmiş *et al.*,  2005*a*, 2005b; Sari *et al.*,  2002, 2004;Çukurovali *et al.*,  2005; Dinçer *et al.*,  2004).

The four-atom bridge O2/C16/C15/C11 linking the cyclobutane and phenoxy rings is planar (Fig. 1). The torsion angle of O2—C16—C15—C11 is -1.0 (1)°. It corresponds to the (-) synclinical configuration. The cyclobutane ring is similar puckered as in a related compounds; the C9/C7/C10 plane forms a dihedaral angle of 24.97 (9)° with the C10/C11/C9 plane in the title compound. The same dihedral angles are presented in the literature: 25.74 (6)° - Çukurovali *et al.*, (2005) and 19.8 (3)° - Dinçer *et al.*, (2004). The mesityl and phenoxy rings are planar. The dihedral angle between these ring is 73.90 (5)°. The maximum deviation from mean plane of the atoms for mesityl and phenoxy rings are -0.046 (6)Å for C5 and 0.016 (3)Å for C17, respectively.

There is one intermolcular non-classical C—H···O hydrogen-bonding interactions in the crystal structure. This interactions lead to close packing between the neighbouring molecules. And the structure is stabilized by van der Waals interactions and symmetry-related molecules are linked to form dimerization chains *via* C—H···O intermolecular non-classical hydrogen bond. Intermolecular van der Waals interactions between the methylcyclobutyl CH group and the O atom of the phenoxy group (Fig. 2, Table 1).

In the IR spectra of 1-(3-mesityl-3-methylcyclobutyl)-2-phenoxy-1-ethanone very important bonds were observed as (ν, cm^-1^); 1711 (sharp C═O stretching) respectively and between 3063–2852 (broad aliphatic and aromatic C—H stretching) and 1070 (ether C–O–C stretching). The ^1^H-NMR spectra were reported in p.p.m. (δ) relative to tetramethylsilane (Si*Me*_4_) as the internal standard and referenced to deuterochloroform (CDCl_3_). In the ^1^H-NMR spectra were H signals obtained as (p.p.m.) 1.7 (s, 3H, cyclobutane-CH_3_), 2.2 (s, 9H, CH_3_-mesitylene), 2.5–2.9 (m, 4H, cyclobutane CH_2_), 3.9 (p,1*H*, cyclobutane CH), 4.2 (s, 2H, O—CH_2_) and aromatic protons observed at 6.7–7.3 (m, 7H, *Ar*H). Elemental Analysis Calc. for C_22_H_26_O_2_ (322.44 g mol^-1^): C, 81.95; H, 8.13 Found: C, 81.88; H, 8.05.

### Experimental

Elemental analysis were determined on a LECO CHNS–932 auto elemental analysis apparatus. Infrared spectra were obtained by using a Mattson 1000 F T–IR Spectrometer, from 4000–400 cm^-1^ in KBr pellet. ^1^H-NMR spectra were recorded on a Jeol FX–90Q Spectrometer 90 MHz.

Synthesis of 1-(3-mesityl-3-methylcyclobutyl)-2-phenoxy-1-ethanone: a mixture of 1-mesityl-1-methyl-3-(2-chloro-1-oxoethyl)cyclobutane (2.64 g, 10 mmol), phenol (1.035 g, 11 mmol) and K_2_CO_3_ (1.51 g, 11 mmol) in 200 ml dry acetone was refluxed for 8 h. The reaction mixture was poured into water (500 ml), the insoluble portion was filtered off, washed with water and crystallized from ethanol (yield 85%).

### Refinement

All H atoms were placed geometrically (C—H = 0.95–0.99 Å) and refined in the riding model approximation, with *U*_iso_(H) = 1.2*U*_eq_(C) for CH and CH_2_, and *U*_iso_(H) = 1.5*U*_eq_(C) for CH_3_.

### Crystal data

**Table:**

C_22_H_26_O_2_	*Z* = 2
*M**_r_* = 322.43	*F*(000) = 348
Triclinic, *P*1	*D*_x_ = 1.200 Mg m^−^^3^
Hall symbol: -P 1	Mo *K*α radiation, λ = 0.71073 Å
*a* = 8.5884 (12) Å	Cell parameters from 149 reflections
*b* = 10.1725 (11) Å	θ = 6.0–20.0°
*c* = 11.1018 (12) Å	µ = 0.08 mm^−^^1^
α = 82.364 (4)°	*T* = 100 K
β = 68.170 (3)°	Block, colourless
γ = 86.235 (2)°	0.42 × 0.33 × 0.24 mm
*V* = 892.24 (19) Å^3^	

### Data collection

**Table:**

Nonius KappaCCD diffractometer	3921 independent reflections
Radiation source: fine-focus sealed tube	2968 reflections with *I* > 2σ(*I*)
graphite	*R*_int_ = 0.054
Detector resolution: 9 pixels mm^-1^	θ_max_ = 27.1°, θ_min_ = 3.0°
ω–scans with 2.00° and 40 sec per frame	*h* = −11→11
Absorption correction: multi-scan (*SADABS*; Bruker–Nonius, 2002)	*k* = −13→12
*T*_min_ = 0.969, *T*_max_ = 0.982	*l* = −14→14
23641 measured reflections	

### Refinement

**Table:**

Refinement on *F*^2^	Primary atom site location: structure-invariant direct methods
Least-squares matrix: full	Secondary atom site location: difference Fourier map
*R*[*F*^2^ > 2σ(*F*^2^)] = 0.047	Hydrogen site location: inferred from neighbouring sites
*wR*(*F*^2^) = 0.129	H-atom parameters constrained
*S* = 1.07	*w* = 1/[σ^2^(*F*_o_^2^) + (0.0644*P*)^2^ + 0.2801*P*] where *P* = (*F*_o_^2^ + 2*F*_c_^2^)/3
3921 reflections	(Δ/σ)_max_ = 0.001
217 parameters	Δρ_max_ = 0.32 e Å^−^^3^
0 restraints	Δρ_min_ = −0.25 e Å^−^^3^

### Special details

**Table:**

Geometry. All s.u.'s (except the s.u. in the dihedral angle between two l.s. planes) are estimated using the full covariance matrix. The cell s.u.'s are taken into account individually in the estimation of s.u.'s in distances, angles and torsion angles; correlations between s.u.'s in cell parameters are only used when they are defined by crystal symmetry. An approximate (isotropic) treatment of cell s.u.'s is used for estimating s.u.'s involving l.s. planes.
Refinement. Refinement of *F*^2^ against ALL reflections. The weighted *R*-factor *wR* and goodness of fit *S* are based on *F*^2^, conventional *R*-factors *R* are based on *F*, with *F* set to zero for negative *F*^2^. The threshold expression of *F*^2^ > σ(*F*^2^) is used only for calculating *R*-factors(gt) *etc*. and is not relevant to the choice of reflections for refinement. *R*-factors based on *F*^2^ are statistically about twice as large as those based on *F*, and *R*-factors based on ALL data will be even larger.

### Fractional atomic coordinates and isotropic or equivalent isotropic displacement parameters (Å^2^)

**Table:**

	*x*	*y*	*z*	*U*_iso_*/*U*_eq_	
C1	0.66157 (19)	0.63782 (15)	0.69324 (15)	0.0191 (3)	
C2	0.6658 (2)	0.62422 (15)	0.81884 (15)	0.0212 (3)	
H2	0.5724	0.5864	0.8899	0.025*	
C3	0.8017 (2)	0.66400 (15)	0.84382 (15)	0.0216 (3)	
C4	0.9374 (2)	0.71600 (15)	0.73790 (15)	0.0202 (3)	
H4	1.0328	0.7416	0.7525	0.024*	
C5	0.93938 (19)	0.73229 (15)	0.61020 (15)	0.0181 (3)	
C6	0.79712 (19)	0.69657 (14)	0.58680 (15)	0.0168 (3)	
C7	0.79622 (18)	0.71572 (15)	0.44807 (15)	0.0175 (3)	
C8	0.8730 (2)	0.59338 (16)	0.37804 (16)	0.0238 (4)	
H8A	0.8710	0.6069	0.2895	0.036*	
H8B	0.8079	0.5150	0.4267	0.036*	
H8C	0.9892	0.5802	0.3731	0.036*	
C9	0.86927 (19)	0.84742 (16)	0.36088 (15)	0.0190 (3)	
H9A	0.8701	0.9204	0.4114	0.023*	
H9B	0.9806	0.8360	0.2918	0.023*	
C10	0.62618 (19)	0.75748 (15)	0.42989 (15)	0.0189 (3)	
H10B	0.5741	0.6854	0.4069	0.023*	
H10A	0.5440	0.7998	0.5038	0.023*	
C11	0.72371 (19)	0.85766 (15)	0.31115 (15)	0.0181 (3)	
H11	0.7558	0.8179	0.2273	0.022*	
C12	0.5091 (2)	0.58448 (17)	0.67897 (17)	0.0255 (4)	
H12A	0.5442	0.5430	0.5980	0.038*	
H12B	0.4298	0.6575	0.6761	0.038*	
H12C	0.4549	0.5185	0.7536	0.038*	
C13	0.8009 (2)	0.64947 (18)	0.98136 (16)	0.0283 (4)	
H13A	0.7400	0.5695	1.0308	0.042*	
H13B	0.7456	0.7273	1.0240	0.042*	
H13C	0.9166	0.6421	0.9784	0.042*	
C14	1.09987 (19)	0.78228 (18)	0.50263 (16)	0.0233 (4)	
H14A	1.1277	0.7295	0.4294	0.035*	
H14B	1.1912	0.7741	0.5361	0.035*	
H14C	1.0846	0.8756	0.4727	0.035*	
C15	0.63834 (19)	0.98988 (16)	0.30363 (15)	0.0196 (3)	
C16	0.4891 (2)	1.00148 (15)	0.26088 (16)	0.0217 (3)	
H16A	0.3888	1.0322	0.3314	0.026*	
H16B	0.5121	1.0673	0.1824	0.026*	
C17	0.35862 (19)	0.87125 (16)	0.16126 (15)	0.0193 (3)	
C18	0.2842 (2)	0.98135 (16)	0.11452 (16)	0.0231 (4)	
H18	0.2968	1.0674	0.1337	0.028*	
C19	0.1910 (2)	0.96324 (19)	0.03910 (18)	0.0302 (4)	
H19	0.1404	1.0381	0.0062	0.036*	
C20	0.1703 (2)	0.8385 (2)	0.01108 (17)	0.0304 (4)	
H20	0.1088	0.8280	−0.0425	0.036*	
C21	0.2405 (2)	0.72904 (18)	0.06224 (18)	0.0290 (4)	
H21	0.2240	0.6427	0.0459	0.035*	
C22	0.3340 (2)	0.74507 (17)	0.13673 (17)	0.0253 (4)	
H22	0.3819	0.6697	0.1714	0.030*	
O1	0.68046 (15)	1.08915 (12)	0.33319 (12)	0.0270 (3)	
O2	0.45853 (14)	0.87559 (11)	0.23207 (11)	0.0235 (3)	

### Atomic displacement parameters (Å^2^)

**Table:**

	*U*^11^	*U*^22^	*U*^33^	*U*^12^	*U*^13^	*U*^23^
C1	0.0195 (8)	0.0117 (7)	0.0253 (8)	−0.0002 (6)	−0.0076 (6)	−0.0015 (6)
C2	0.0221 (8)	0.0149 (8)	0.0224 (8)	−0.0030 (6)	−0.0038 (7)	0.0004 (6)
C3	0.0289 (9)	0.0143 (7)	0.0217 (8)	−0.0001 (6)	−0.0088 (7)	−0.0040 (6)
C4	0.0214 (8)	0.0183 (8)	0.0239 (8)	−0.0011 (6)	−0.0104 (7)	−0.0058 (6)
C5	0.0179 (7)	0.0156 (7)	0.0211 (8)	0.0010 (6)	−0.0066 (6)	−0.0056 (6)
C6	0.0179 (7)	0.0129 (7)	0.0203 (7)	0.0013 (6)	−0.0069 (6)	−0.0047 (6)
C7	0.0159 (7)	0.0175 (8)	0.0198 (7)	−0.0008 (6)	−0.0066 (6)	−0.0043 (6)
C8	0.0258 (8)	0.0229 (8)	0.0263 (8)	0.0048 (7)	−0.0122 (7)	−0.0099 (7)
C9	0.0168 (7)	0.0216 (8)	0.0177 (7)	−0.0018 (6)	−0.0047 (6)	−0.0035 (6)
C10	0.0174 (7)	0.0177 (8)	0.0237 (8)	−0.0006 (6)	−0.0094 (6)	−0.0039 (6)
C11	0.0187 (7)	0.0190 (8)	0.0182 (7)	−0.0002 (6)	−0.0078 (6)	−0.0048 (6)
C12	0.0228 (8)	0.0225 (9)	0.0302 (9)	−0.0085 (7)	−0.0104 (7)	0.0061 (7)
C13	0.0371 (10)	0.0267 (9)	0.0210 (8)	−0.0066 (8)	−0.0103 (7)	−0.0013 (7)
C14	0.0171 (8)	0.0321 (9)	0.0218 (8)	−0.0037 (7)	−0.0072 (7)	−0.0049 (7)
C15	0.0203 (8)	0.0197 (8)	0.0174 (7)	−0.0029 (6)	−0.0048 (6)	−0.0026 (6)
C16	0.0219 (8)	0.0167 (8)	0.0282 (8)	0.0012 (6)	−0.0099 (7)	−0.0070 (7)
C17	0.0148 (7)	0.0211 (8)	0.0218 (8)	0.0001 (6)	−0.0062 (6)	−0.0036 (6)
C18	0.0218 (8)	0.0190 (8)	0.0277 (8)	−0.0038 (6)	−0.0093 (7)	0.0018 (7)
C19	0.0283 (9)	0.0314 (10)	0.0328 (9)	−0.0060 (7)	−0.0169 (8)	0.0095 (8)
C20	0.0268 (9)	0.0434 (11)	0.0240 (9)	−0.0084 (8)	−0.0121 (7)	−0.0023 (8)
C21	0.0248 (9)	0.0301 (9)	0.0351 (10)	0.0000 (7)	−0.0106 (8)	−0.0154 (8)
C22	0.0215 (8)	0.0204 (8)	0.0365 (9)	0.0040 (7)	−0.0124 (7)	−0.0081 (7)
O1	0.0317 (7)	0.0214 (6)	0.0334 (7)	−0.0009 (5)	−0.0166 (6)	−0.0082 (5)
O2	0.0276 (6)	0.0151 (6)	0.0350 (6)	0.0000 (5)	−0.0196 (5)	−0.0036 (5)

### Geometric parameters (Å, °)

**Table:**

C1—C2	1.397 (2)	C12—H12A	0.9800
C1—C6	1.410 (2)	C12—H12B	0.9800
C1—C12	1.518 (2)	C12—H12C	0.9800
C2—C3	1.392 (2)	C13—H13A	0.9800
C2—H2	0.9500	C13—H13B	0.9800
C3—C4	1.385 (2)	C13—H13C	0.9800
C3—C13	1.512 (2)	C14—H14A	0.9800
C4—C5	1.399 (2)	C14—H14B	0.9800
C4—H4	0.9500	C14—H14C	0.9800
C5—C6	1.418 (2)	C15—O1	1.2177 (19)
C5—C14	1.514 (2)	C15—C16	1.516 (2)
C6—C7	1.529 (2)	C16—O2	1.4237 (18)
C7—C8	1.535 (2)	C16—H16A	0.9900
C7—C9	1.562 (2)	C16—H16B	0.9900
C7—C10	1.571 (2)	C17—O2	1.3682 (18)
C8—H8A	0.9800	C17—C18	1.389 (2)
C8—H8B	0.9800	C17—C22	1.392 (2)
C8—H8C	0.9800	C18—C19	1.391 (2)
C9—C11	1.536 (2)	C18—H18	0.9500
C9—H9A	0.9900	C19—C20	1.383 (3)
C9—H9B	0.9900	C19—H19	0.9500
C10—C11	1.553 (2)	C20—C21	1.387 (3)
C10—H10B	0.9900	C20—H20	0.9500
C10—H10A	0.9900	C21—C22	1.380 (2)
C11—C15	1.497 (2)	C21—H21	0.9500
C11—H11	1.0000	C22—H22	0.9500
			
C2—C1—C6	119.74 (14)	C10—C11—H11	111.2
C2—C1—C12	116.96 (14)	C1—C12—H12A	109.5
C6—C1—C12	123.28 (14)	C1—C12—H12B	109.5
C3—C2—C1	122.43 (15)	H12A—C12—H12B	109.5
C3—C2—H2	118.8	C1—C12—H12C	109.5
C1—C2—H2	118.8	H12A—C12—H12C	109.5
C4—C3—C2	117.38 (14)	H12B—C12—H12C	109.5
C4—C3—C13	121.65 (15)	C3—C13—H13A	109.5
C2—C3—C13	120.97 (15)	C3—C13—H13B	109.5
C3—C4—C5	122.47 (15)	H13A—C13—H13B	109.5
C3—C4—H4	118.8	C3—C13—H13C	109.5
C5—C4—H4	118.8	H13A—C13—H13C	109.5
C4—C5—C6	119.57 (14)	H13B—C13—H13C	109.5
C4—C5—C14	116.94 (14)	C5—C14—H14A	109.5
C6—C5—C14	123.43 (13)	C5—C14—H14B	109.5
C1—C6—C5	118.27 (14)	H14A—C14—H14B	109.5
C1—C6—C7	121.45 (13)	C5—C14—H14C	109.5
C5—C6—C7	120.21 (13)	H14A—C14—H14C	109.5
C6—C7—C8	110.39 (12)	H14B—C14—H14C	109.5
C6—C7—C9	117.38 (12)	O1—C15—C11	123.12 (14)
C8—C7—C9	111.83 (13)	O1—C15—C16	117.80 (14)
C6—C7—C10	118.07 (12)	C11—C15—C16	119.06 (13)
C8—C7—C10	110.26 (12)	O2—C16—C15	109.41 (13)
C9—C7—C10	87.08 (11)	O2—C16—H16A	109.8
C7—C8—H8A	109.5	C15—C16—H16A	109.8
C7—C8—H8B	109.5	O2—C16—H16B	109.8
H8A—C8—H8B	109.5	C15—C16—H16B	109.8
C7—C8—H8C	109.5	H16A—C16—H16B	108.2
H8A—C8—H8C	109.5	O2—C17—C18	124.76 (14)
H8B—C8—H8C	109.5	O2—C17—C22	115.14 (14)
C11—C9—C7	89.84 (11)	C18—C17—C22	120.10 (15)
C11—C9—H9A	113.7	C17—C18—C19	118.77 (16)
C7—C9—H9A	113.7	C17—C18—H18	120.6
C11—C9—H9B	113.7	C19—C18—H18	120.6
C7—C9—H9B	113.7	C20—C19—C18	121.37 (17)
H9A—C9—H9B	110.9	C20—C19—H19	119.3
C11—C10—C7	88.89 (11)	C18—C19—H19	119.3
C11—C10—H10B	113.8	C19—C20—C21	119.17 (16)
C7—C10—H10B	113.8	C19—C20—H20	120.4
C11—C10—H10A	113.8	C21—C20—H20	120.4
C7—C10—H10A	113.8	C22—C21—C20	120.27 (16)
H10B—C10—H10A	111.1	C22—C21—H21	119.9
C15—C11—C9	117.93 (13)	C20—C21—H21	119.9
C15—C11—C10	114.89 (13)	C21—C22—C17	120.25 (16)
C9—C11—C10	88.64 (11)	C21—C22—H22	119.9
C15—C11—H11	111.2	C17—C22—H22	119.9
C9—C11—H11	111.2	C17—O2—C16	118.20 (12)

### Hydrogen-bond geometry (Å, °)

**Table:**

*D*—H···*A*	*D*—H	H···*A*	*D*···*A*	*D*—H···*A*
C10—H10A···O1^i^	0.99	2.44	3.419 (3)	172 (2)

Symmetry codes: (i) −*x*+1, −*y*+2, −*z*+1.
